# Novel Antioxidant Peptides from Crassostrea Hongkongensis Improve Photo-Oxidation in UV-Induced HaCaT Cells

**DOI:** 10.3390/md20020100

**Published:** 2022-01-24

**Authors:** Chen Zhang, Jiatong Lv, Xiaoming Qin, Zhilan Peng, Haisheng Lin

**Affiliations:** 1 College of Food Science and Technology, Guangdong Ocean University, Zhanjiang 524088, China; 18854145522@163.com (C.Z.); jiatonglv@126.com (J.L.); pengzhilan@stu.gdou.edu.cn (Z.P.); linhs@gdou.edu.cn (H.L.); 2 Guangdong Provincial Key Laboratory of Aquatic Product Processing and Safety, Zhanjiang 524088, China; 3 National Research and Development Branch Center for Shellfish Processing (Zhanjiang), Zhanjiang 524088, China; 4 Guangdong Province Engineering Laboratory for Marine Biological Products, Zhanjiang 524088, China; 5 Guangdong Provincial Engineering Technology Research Center of Marine Food, Zhanjiang 524088, China; 6 Collaborative Innovation Center of Seafood Deep Processing, Dalian Polytechnic University, Dalian 116034, China

**Keywords:** oyster, peptides, photo-oxidation, HaCaT cells

## Abstract

Enzymatic hydrolysates from Oysters (OAH) display multiple biological activities. Previously, a 3~5 KDa oyster ultrafiltration component (OUP) showed a high property of preventing skin oxidation. Subsequently, we identified specific peptides with such activity. OUP was fractionated stepwise by Sephadex-G25 and RP-HPLC, and active fractions were screened using UV-irradiated HaCaT cells. The most active fractions (OP5-3) were analyzed by LC-MS/MS and a total of 17 peptides were identified. Results from mass spectrometry showed that OP5-3 consisted of peptides with a molecular weight range of 841.51–1786.92 Da. Six of these peptides were synthesized for validating the activity of resisting skin oxidation in the same cell model. All six peptides showed varying degrees of antioxidant activity, while pretreatment of HaCaT cells with AIVAEVNEAAK alleviated UV cytotoxicity, inhibited metalloproteinase 1 (MMP-1) expression, and showed the highest activity to resist UV-induced skin photo-oxidation among these peptides. In addition, results from molecular docking analysis of MMP-1 with AIVAEVNEAAK showed that AIVAEVNEAAK suppresses its enzymatic activity by directly interacting with MMP-1 and thus exhibit anti-photoaging activity.

## 1. Introduction

Skin aging involves both intrinsic chronological aging and extrinsic aging caused by various external stimuli, mainly ultraviolet radiation. This latter process is termed photoaging [1]. Even if the skin can regenerate and repair itself, the intrinsic chronological aging is irreversible, and excessive exposure to ultraviolet rays will aggravate skin aging [2]. Accumulating skin damage increases the incidence of skin cancer, lowers the quality of life, and imposes a financial burden. Therefore, mitigating photoaging is essential if skin health is to be improved and quality of life maintained.

Pathological effects of UV radiation range from erythema and premature aging to cancer. Cellular modifications, such as alterations in elastic fibers and collagen, loss of subcutaneous adipose tissue, and photo-carcinogenic changes, are common [3]. These adverse effects are particularly relevant for epidermal cells, such as keratinocytes, due to their location on the surface of the skin. Epidermal cells are continuously exposed to oxidative stress via reactive oxygen species (ROS) caused by UV radiation. The accumulation of ROS induces overproduction of matrix metalloproteinases (MMPs) that deplete keratinocyte stem cells through cleavage of COL17A1 [4] and degrade the dermal collagen matrix. This process is primarily responsible for UV-irradiation-induced photoaging. Skin photoaging in the general population has recently increased because of the progressive depletion of the ozone layer [5]. Increased awareness of photoaging has led to the development of skin anti-photoaging agents in the pharmaceutical and cosmetic fields.

Significant effort has been expended in the development of multiple active anti-photoaging products, most of which are naturally occurring. These products may be beneficial for maintaining skin health and preventing skin damage and inflammation [6,7]. In particular, antioxidant [3], anti-inflammatory, and antiapoptotic activities help to protect normal skin cells from UVB irradiation; the present study screens for peptides with anti-photoaging activity from enzymatic hydrolysates from oysters (Crassostrea hongkongensis). Oysters are economically important bivalves that inhabit mudflats and are cultivated worldwide. According to the Compendium of Materia Medica written by Li Shizhen, a famous medical scientist in ancient Chinese, dietary oysters supplementation can white and rejuvenate skin. Further, many studies show that oyster and oyster-derived peptides can help treat hyperglycemia [8] and chronic alcohol-induced liver injury [9], improve reproductive ability [10,11], and display anti-apoptotic [12,13], antioxidant [14,15,16,17], immunomodulatory [18], and anti-inflammatory properties [19]. In recent years, a new term “target repositioning” has been proposed, to highlight that druggable protein targets implicated in multiple diseases (hubs in the diseasome) can be exploited to accelerate the discovery of molecularly targeted cosmeceuticals that can promote skin health as an added benefit [20]. Jae Hyeong Han et al. reported anti-melanogenic effects of an oyster hydrolysate in UVB-irradiated C57BL/6J mice and B16F10 melanoma cells. This action was associated with the downregulation of cAMP signaling [21]. Likewise, according to the results of the previous study of our team, Zhilan Peng et al. reported that anti-photoaging effects of an oyster protein enzymatic hydrolysate in UVB-induced photoaging HaCaT cells. This activity was possibly associated with its anti-antioxidant property and ability to enhance extracellular procollagen I expression [22].

However, the chemical nature of oyster-derived against photo-oxidation and anti-photoaging products has received little attention. In this study, we applied an efficient enzymatic method for the preparation of peptides with significant activity from Hong Kong oysters (C. hongkongensis) proteins. The anti-oxidant activity of these peptides was investigated using UV-irradiated human keratinocytes (HaCaT). The activity resisting photo-oxidation was evaluated and peptides were isolated, purified, and identified. Amino acid composition and sequences were also analyzed. Highly active peptides were subsequently synthesized and the effect of preventing skin oxidation was verified.

## 2. Results

### 2.1. Isolation and Screening of Active Fractions from OUP (3~5 kDa)

**Figure 1:**
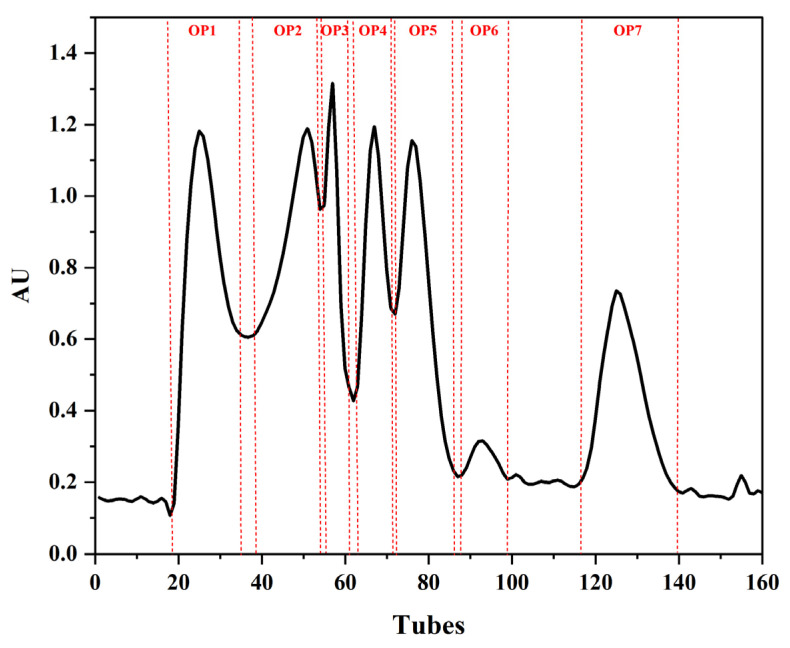
Gel permeation chromatogram of OUP on a Sephadex G-25 column.

OUP from OAH previously showed the best anti-photoaging activity for the back skin of UV-irradiated mice. OUP was thus screened to further isolate peptides with anti-photoaging potential using Sephadex-G25. OUP was separated into seven fractions (OP1~OP7) based on UV (214 nm) absorbance (From Figure 1). Each fraction was collected separately, freeze-dried, and used for subsequent experiments.

Combinations of assays describe the antioxidant properties of peptides in more detail than any single method (Moure, Dominguez, and Parajo, 2006), and the antioxidant activity of each fraction was assessed by DPPH/hydroxyl/ABTS scavenging activities and total protein content. Fractions OP2 and OP3 showed higher DPPH radical scavenging (89.49% and 87.99%, respectively) and higher hydroxyl radical scavenging activity (74.31% and 68.14%, respectively) activities. Fractions OP4 and OP7 displayed higher ABTS radical scavenging activity (26.10% and 36.61%, respectively.) (From Table 1). Results were the same when activities were normalized for total protein. OP1 and OP2 were complex mixtures and OP6 and OP7 showed low yield. Therefore, OP3, OP4, and OP5 were selected for further study. Below, OP3 and OP4, and OP5 are referred to as OPs.

### 2.2. Cell Viability

#### 2.2.1. Viability of HaCaT Cells after UV-Irradiation

**Figure 2:**
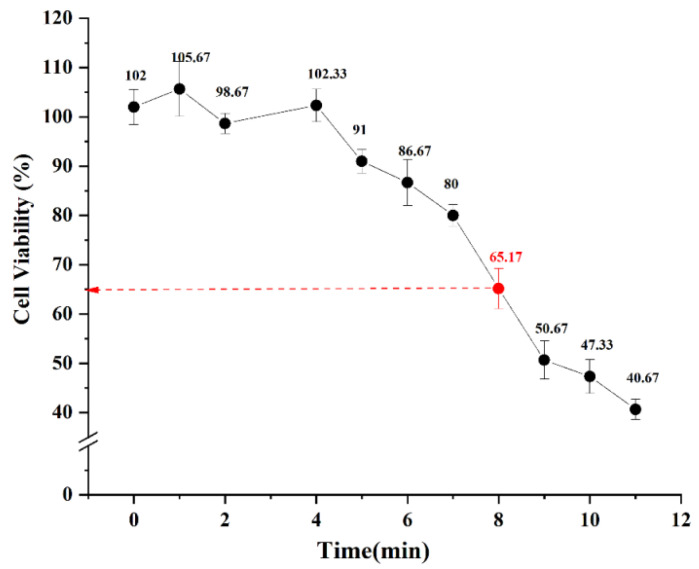
Effect of the time of UV irradiation on cell viability.

UV cytotoxicity for HaCaT cells was evaluated by CCK-8 assay 24 h after irradiation (Figure 2). UV significantly reduced cell viability in a dose-dependent manner. Cell viability was reduced to 66.96% with a UV dose of 31.2 mJ/cm^2^ and decreased to about 45% with a UVB dose of 42.9 mJ/cm^2^. The former dose is a suitable molding condition.

#### 2.2.2. Evaluation of OPs Cytotoxicity

**Figure 3:**
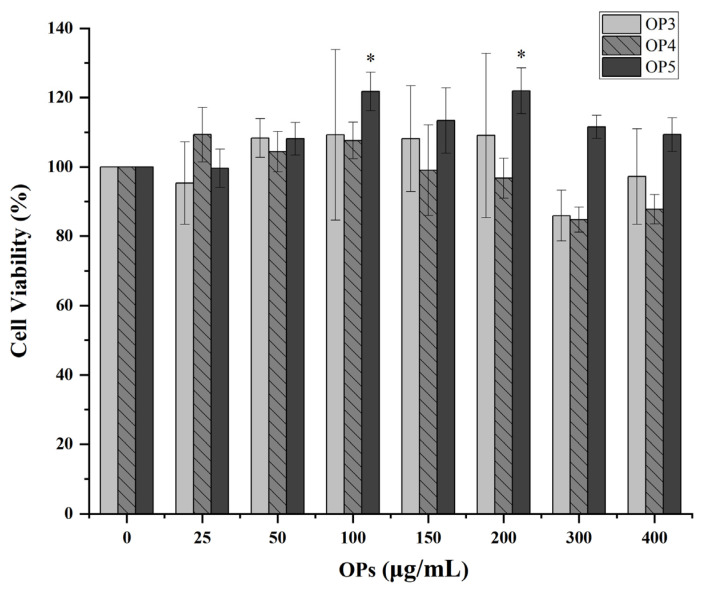
Effect of OPs on cell viability. * *p* < 0.05, compared with untreated cells.

HaCaT cells were treated with different concentrations of OP3, OP4, and OP5 individually in a serum-free medium (0–25–50–100–150–200–300–400 μg/mL) for one day. No decrease in cell viability with respect to basal conditions was observed in cells treated with OP3 and OP5 (Figure 3). Further, no toxicity was observed in cells treated with OP4 and doses up to 200 μg/mL. Thus, we used concentrations of 0–200 μg/mL for subsequent experiments.

#### 2.2.3. Effect of UV-Irradiation on HaCaT Cell Viability after Pretreatment of OPs

**Figure 4:**
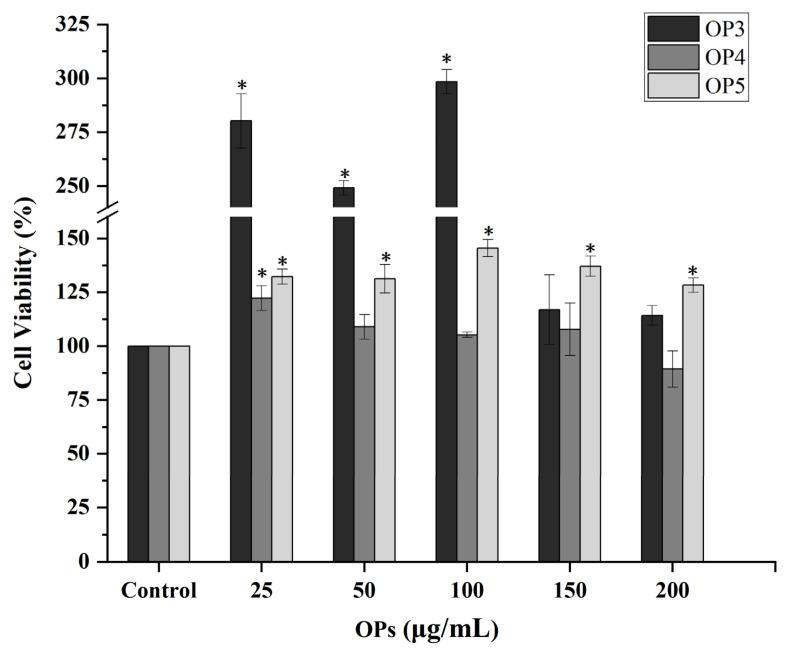
Effect of OPs pretreatment on cell viability of UV-irradiated HaCaT cells. * *p* < 0.05, compared to cells without pretreatments.

HaCaT cells were treated with different concentrations of OP3, OP4, and OP5 individually in a serum-free medium (0–25–50–100–150–200 μg/mL) for one day after 12 h of subculture. Cell viability was assessed by the CCK-8 method after UV-irradiation. All fractions enhanced cell survival (Figure 4). Two concentrations, 25 and 100 μg/mL, were selected as optimal and were used for validation assays.

### 2.3. Inhibition of Reactive Oxygen Species (ROS) Generation by OPs

**Figure 5:**
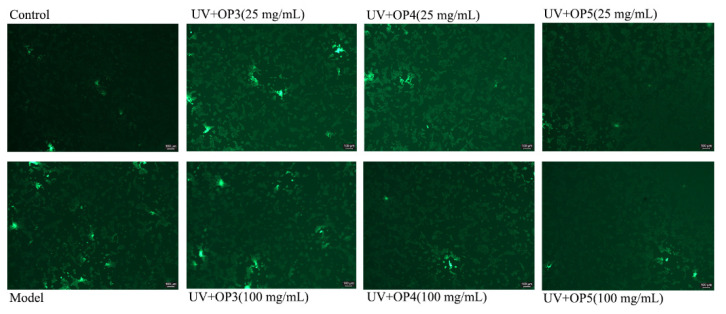
OPs suppress UV-induced ROS production in HaCaT cells.

Overexpression of ROS induced by UV-irradiation causes oxidative stress that damages cells. We investigated the impact of OP3, OP4, and OP5 on ROS levels in UV-exposed HaCaT cells using a fluorescence probe. Fluorescence intensity following UV-irradiation was abnormally strong in HaCaT cells. Cells pretreated with 25, 50, and 100 μg/mL of OPs showed weaker green fluorescence and thus less oxidative stress (Figure 5). Treatment with OP5 caused the greatest effect. Thus, OP5 may be a potent antioxidant t that can protect cells from UV-induced oxidation of cellular biomolecules.

### 2.4. Effects of OPs on Malondialdehyde (MDA) Level and Superoxide Dismutase (SOD) Activity

**Figure 6:**
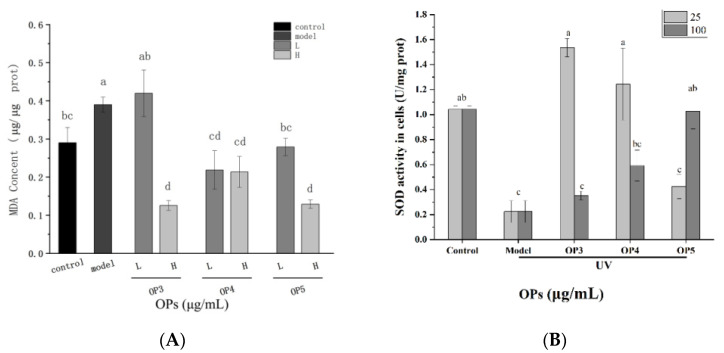
MDA level in HaCaT cells (**A**) and SOD activity in HaCaT cells (**B**). Different letters indicate significant differences between the two, *p* < 0.05.

MDA levels were significantly increased by UV-irradiation and SOD activities were decreased (*p* < 0.05). Pretreatment with OPs induced a reduction of MDA levels in HaCaT cells and an increase in SOD activity (*p* < 0.05) (Figure 6). Further validation, the protective effect of peptides on skin cells was achieved by reducing UV-induced oxidative stress.

### 2.5. β-Galactosidase (SA-β-Gal) Staining

**Figure 7:**
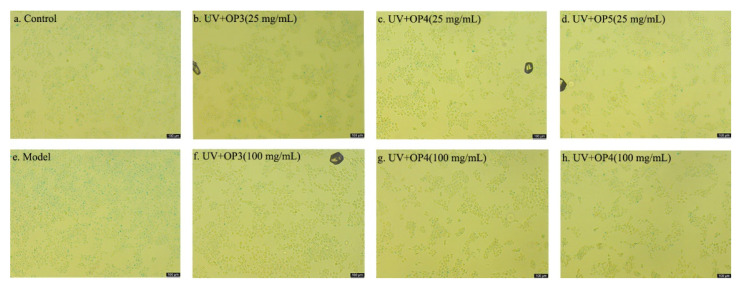
SA-β-gal staining of HaCaT cells treated with UV with and without OP pretreatment. (**a**): normal control, (**e**): only UV, (**b**): UV+ OP3(25 mg/mL), (**f**): UV+ OP3(100 mg/mL), (**c**): UV+ OP4(25 mg/mL), (**g**): UV+ OP4(100 mg/mL), (**d**): UV+ OP5(25 mg/mL), (**h**): UV+ OP5(100 mg/mL).

HaCaT cell senescence induced by UV-irradiation was assessed using senescence-associated SA-*β*-gal staining. UV-irradiated cells show abnormally high percentages of SA-*β*-gal-positive cells compared with unexposed cells. In contrast, 100 mg/mL OP3 or 25 mg/mL OP5 remarkably reduced the numbers of SA-*β*-gal-positive cells compared with UV-irradiated cells (Figure 7).

### 2.6. Effect of Pretreatment for OPs on the Expression of Aminoterminal Propeptide of Type I Procollagen (PINP)

**Figure 8:**
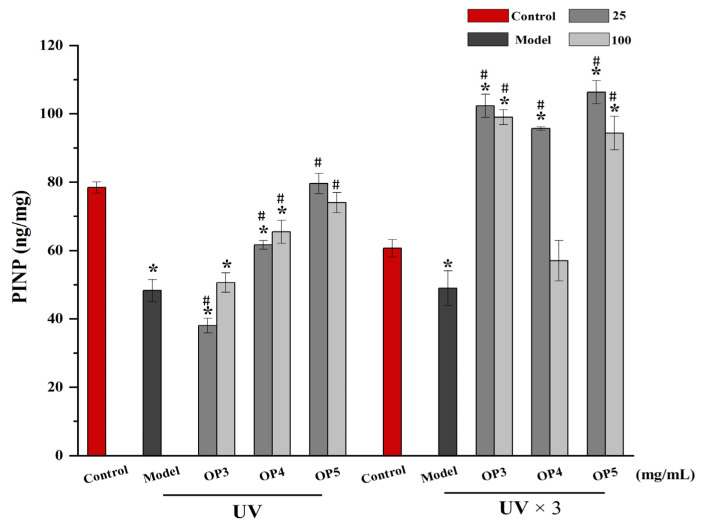
Effect of OPs on the expression of PINP proteins. * *p*< 0.05, compared with control group; # *p* < 0.05, compared with model group.

HaCaT cells are unable to synthesize collagen but can generate PINP, a precursor of Type I collagen. The amount of PINP expression can be used to judge the photoaging of HaCaT cells. UV-irradiated HaCaT cells were pretreated with OPs, and PINP was measured by ELISA assay. The results showed that once and three times of UV-irradiation HaCaT cells, the expression of PINP will decrease significantly, while OPs reversed the impact of UV exposure on PINP levels (Figure 8). Treatment with OP5 (25 μg/mL) displayed the greatest effect. Hence, OPs treatment could exert an anti-photoaging effect on UVB-irradiated HaCaT cells by lessening PINP downregulation.

### 2.7. Purification of OP5 by RT-HPLC

**Figure 9:**
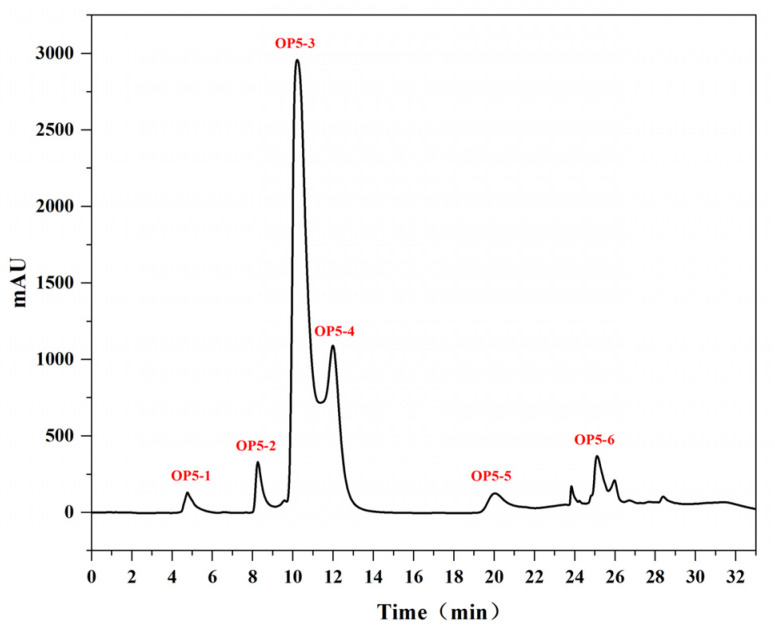
C18 RP-HPLC chromatogram of OP5.

**Figure 10:**
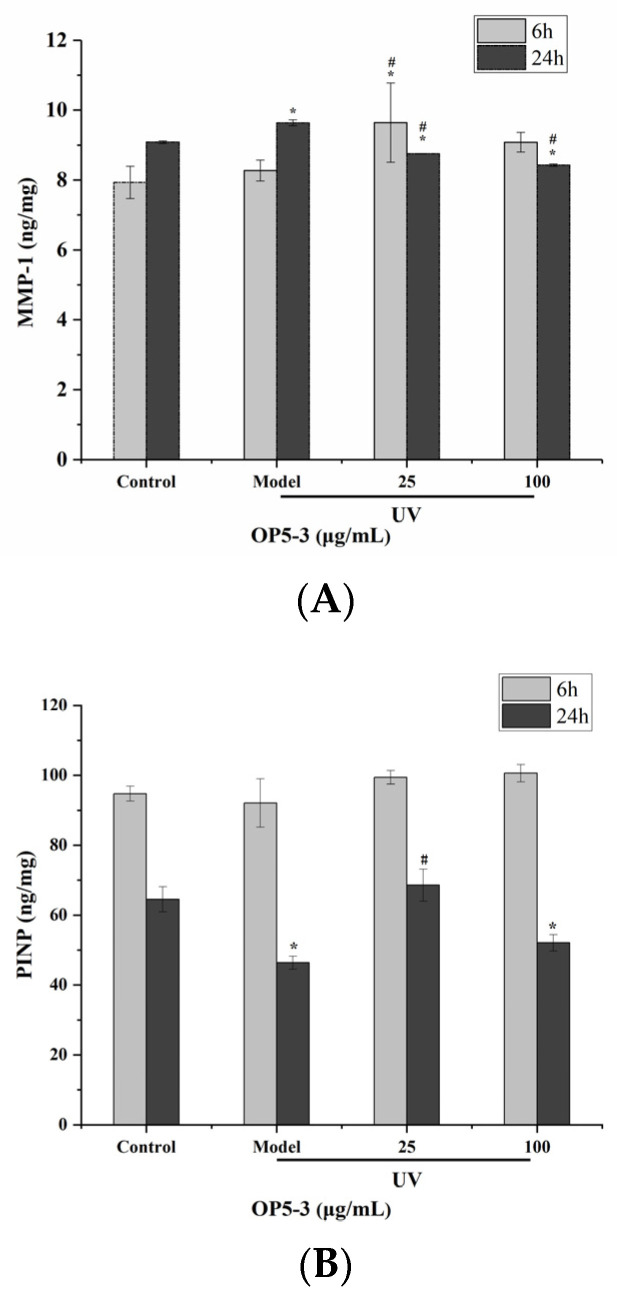
Effect of OP5-3 on the expression of MMP-1 (**A**) and PINP (**B**) proteins. * *p* < 0.05, compared with control group; # *p* < 0.05, compared with model group.

The cell study identified peptide fractions that display the effect of inhibiting photo-oxidation on UV-irradiated HaCaT cells. OP5 was the most potent fraction and was further purified by reverse-phase liquid chromatography, using an Xbridge C18 RP-HPLC column, which separates peptides based on hydrophobicity. Six major peaks were identified (Figure 9), and peptide content was highest in the third peak (OP5-3; Figure 10). We selected OP5-3 for structural identification, considering sample size, experimental sustainability, and later analyses.

### 2.8. Effect of Pretreatment with OP5-3 on the Expressions of PINP and MMP-1

MAPK activation induced by UV-irradiation contributes to the upregulation of MMPs, especially MMP-1, leading to ECM degradation, including collagen and elastin. PINP and MMP-1 expression were measured by ELISA assay, at 6 and 24 h after irradiation. In general, expression of PINP and MMP-1 at 6 h after irradiation were not significantly different from controls (results not shown). However, at 24 h after irradiation, PINP expression was decreased, and MMP-1 expression was significantly increased after UV exposure. Pretreatment of HaCaT cells with OP5-3 reversed this reduction in PINP and lowered MMP-1 expression (Figure 10).

### 2.9. The Main Peptide Sequences of OP5-3 Were Identified by Mass Spectroscopy

**Figure 11:**
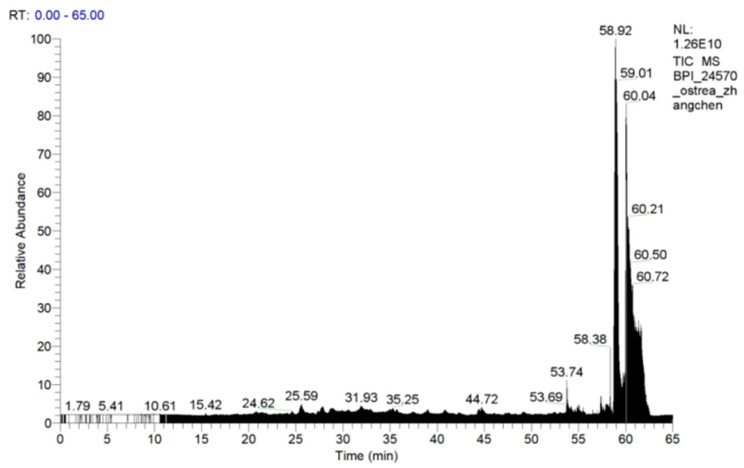
Total ion chromatogram of OP5-3.

Peptide fingerprinting of 17 characteristic OPs used liquid chromatography-mass spectrometry (LC-MS/MS) (Figure 11). Molecular weights of OPs ranged from 841.5095 to 1933.9066 Da (amino acid residues 7–17) (Table 2). Due to cost and time constraints, we selected six peptides, based on protein scores and hydrophobic amino acid ratios in their mass spectra, for synthesis and activity validation (Table 3).

### 2.10. Verification of the Anti-Photoaging Activity of Synthetic Peptides in HaCaT Cells

**Figure 12:**
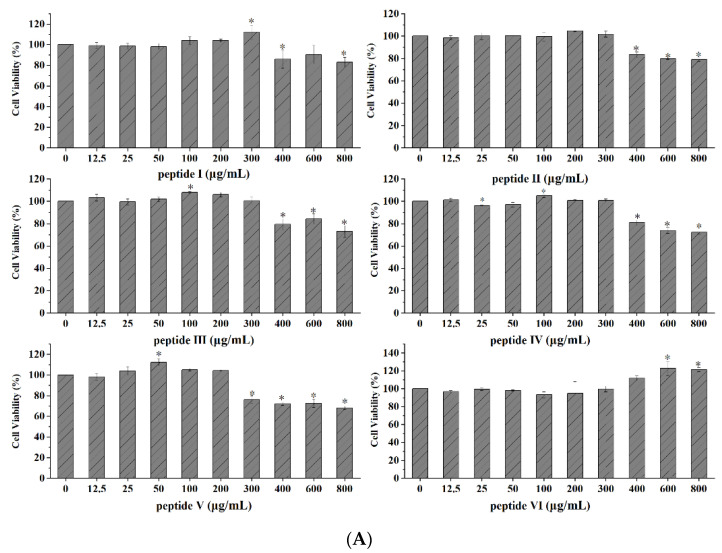

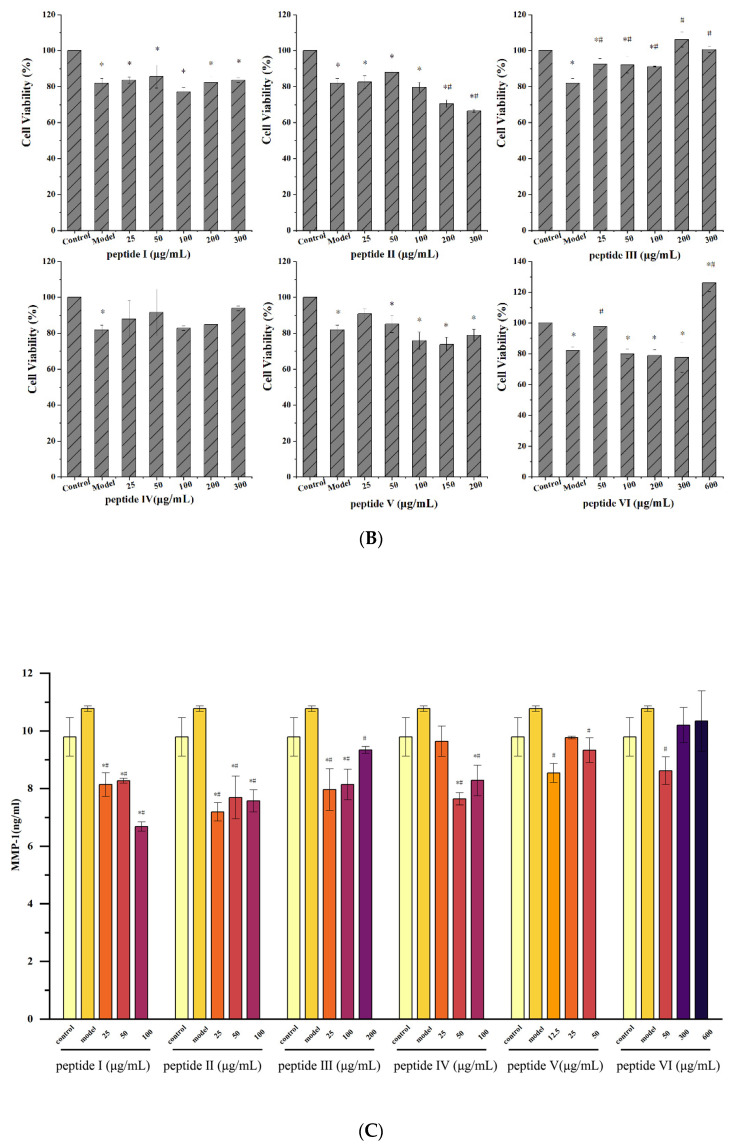
Verification of inhibiting photo-oxidation activity of synthetic peptides in HaCaT cells. (**A**) Effect of synthetic peptides on cell viability; (**B**) Effect of synthetic peptides on cell viability of UV-irradiated HaCaT cells; (**C**) Effect of synthetic peptides on the expression of MMP-1. * *p* < 0.05, compared with control group; # *p* < 0.05, compared with model group.

Cytotoxicity and optimal concentrations of synthetic peptides and expression of MMP-1 were described in methods. Peptide I to IV showed no cytotoxicity at concentrations from 0 to 300 μg/mL. Similarly, Peptide V showed no toxicity at 0 to 200 μg/mL, and Peptide VI showed none at 0 to 600 μg/mL (Figure 12A). Peptides III and VI showed beneficial effects on the survival of UV-irradiated HaCaT cells, but the other four peptides did not (Figure 12B). Finally, all synthetic peptides inhibited the abnormal upregulation of MMP-1 to varying degrees. Peptide I displayed the most prominent effect (Figure 12C). This peptide, AIVAEVNEAAK, and MMP-1 were further assessed using molecular docking.

### 2.11. Molecular Docking Analysis of MMP-1 with AIVAEVNEAAK

**Figure 13:**
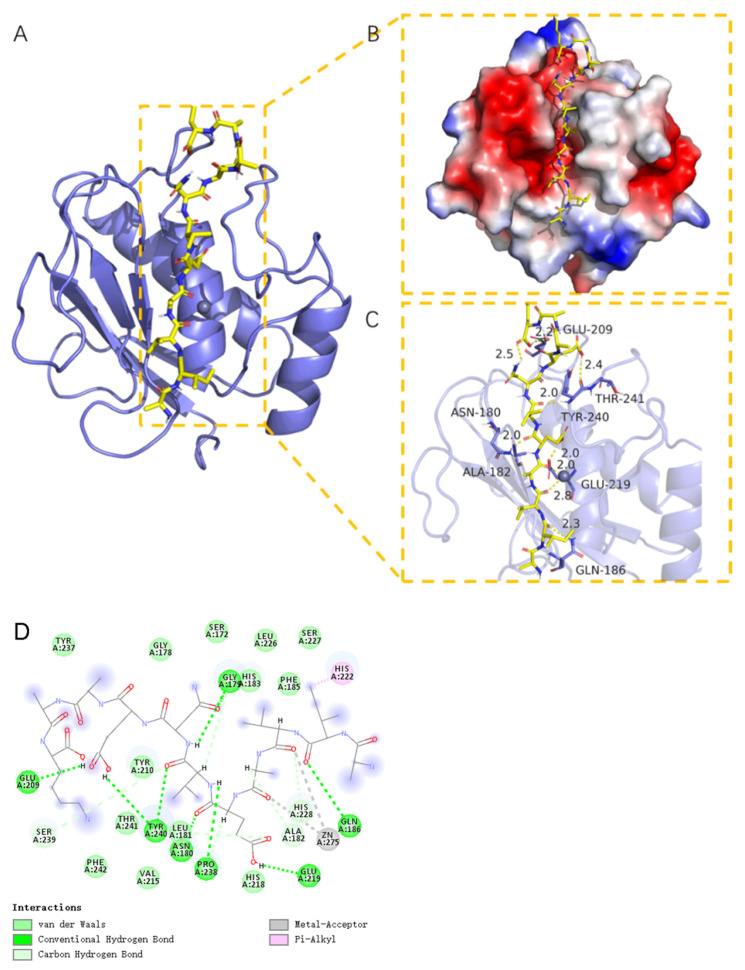
The binding of AIVAEVNEAAK with MMP-1 protein. (**A**) The 3D structure of the complex. (**B**) The surface of the active site. (**C**) Detail binding within the complex. The backbone of protein is rendered as tube and colored blue. The peptide is rendered yellow. Yellow dashes represent hydrogen bond distances. (**D**) 2D diagram of the interaction between AIVAEVNEAAK and amino acid residues of MMP-1.

AIVAEVNEAAK was examined as a ligand for docking with MMP-1 protein. AIVAEVNEAAK binds well with the target protein with a high degree of matching; binding energy is less than −6 kcal/mol (Figure 13A). The complex formed by the docked peptide and target protein was visualized using Pymol 2.1 software (PyMOL 2.1, Schrödinger Inc., New York, NY, USA) to identify binding modes. Amino acid residues involved in binding at the active site include LYS-771, GLU-802, PHE-649, PHE-1013, SER-876, THR-1010, GLU-89, and HIS-875. AIVAEVNEAAK forms multiple hydrogen bonds with several amino acids (LYS-771, GLU-802, THR-1010, GLU-879, and co-form SER-876) with short hydrogen bond distances and strong binding forces. These interactions stabilize peptide ligands at the active site. In addition, the surface of the active site for the target protein (Figure 13B) illustrates the significant match between ligand and pocket.

### 2.12. ESI-MS and MS/MS Spectrum of the Peptide AIVAEVNEAAK

**Figure 14:**
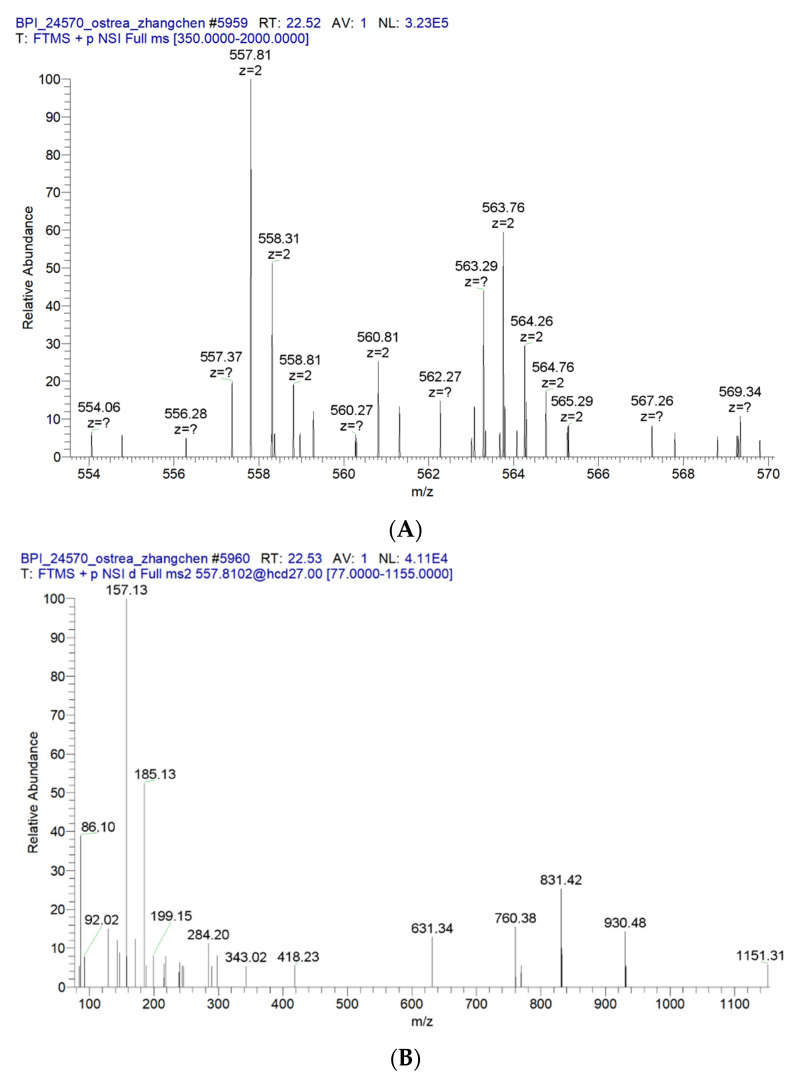
ESI-MS (**A**) and MS/MS (**B**) spectrum of the peptide AIVAEVNEAAK.

We analyzed OP5-3 by mass spectrometry, obtained 17 peptide sequences, and verified six of them. Only the primary and secondary mass spectrometry of AIVAEVNEAAK with the most potent activity is provided (Figure 14A,B).

## 3. Discussion

Manifestations of skin photoaging include irreversible loss of skin moisture, increased skin folding, skin thickening, greatly reduced elasticity, and skin-related cancer. Currently, UV light is believed to induce excessive ROS production in skin cells. Continuous skin exposure to UV can lead to ROS accumulation, with accompanying oxidation of matrix and macromolecules, such as DNA, RNA, and proteins. Extensive damage to cytogenetic material may be irreparable and can trigger the automatic removal of cells via apoptosis [23,24].

Recently, marine organisms have received increasing attention as sources of active compounds in foods and pharmaceutical raw materials [25]. Oysters are reported to produce chemicals with a variety of biological activities. These bivalves represent a pool of active raw materials. Further, the animals are a major cultured shellfish worldwide, and its entire genome has been sequenced [26]. Some anti-photoaging peptides have been isolated and identified in oysters and most active peptides are small, generally with several to a dozen amino acid residues. Therefore, this study used enzyme hydrolysis to treat oyster tissues to obtain peptides. Four fractions were obtained by grading enzymatic products, and OP1 to OP7 components from the highest 3~5 KDa components were separated by Sephadex-G25. OP1 and OP2 components were complex, which posed problems for subsequent analysis. OP6 and OP7 components were mainly amino acids. Thus, OP3, OP4, and OP5 were selected for assessing biological activity.

The molding time of UV-irradiation was 8 min and the total irradiation dose was 31.2 mJ/cm^2^ (UVA_365 nm_ = 28.8 mJ/cm^2^, UVB_310 nm_ = 2.4 mJ/cm^2^). No significant change in cell survival was observed immediately after irradiation, but cell survival was reduced to about 65% compared with controls 24h after irradiation. We believe that UV might cause apoptosis and the toxic effect of UV-irradiation on cells is somewhat delayed. Cell viability was determined using CCK-8 assays and showed that pretreatment with 25–100 μg/mL OP3, 25 μg/mL OP4, and 25–200 μg/mL OP5 alleviated the toxic effect of UV on HaCaT cells.

The human body can adapt to many environments. Various environmental factors can cause an abnormal increase in ROS and the accumulation of free radicals. Often, antioxidant enzymes can remove free radicals and avoid significant damage to skin cells. However, ROS production can exceed cellular antioxidant capacity thus disrupting the intracellular antioxidant enzyme system and damaging important cellular components, such as DNA, proteins, lipid membranes, and mitochondria [27]. We confirmed that 24h after UV exposure SOD activity was significantly reduced and ROS and MDA significantly increased. These effects could be substantially ameliorated by pretreatment of HaCaT cells with OPs. SOD activity increased, indicating restoration of antioxidant capacity, and MDA content was reduced indicating reduced levels of that intracellular lipid peroxide. Hence, OPs showed antioxidant effects, consistent with the results of Wu et al. [15].

Normal cells can lose their ability to divide due to intrinsic factors or extrinsic induction and enter a state of senescence. In this state, cells survive but vary greatly in expression profiles of cellular genes and proteins [28,29,30,31,32,33]. Particularly, β-galactosidase, which exhibits high activity, is a marker for senescent cells [34]. β-galactosidase, using X-Gal as a substrate, generates dark blue substances. Blue staining produced by aging-specific β-galactosidase can be directly observed and cells counted under an ordinary optical microscope. The percentage of β-galactosidase positive cells after UV-irradiation was significantly increased compared with control cells. Conversely, the percentage of β-galactosidase positive cells was significantly decreased compared with UV-irradiation cells after pretreatment with 100 μg/mL OP3 or 25 μg/mL OP5. These findings were consistent with the effect of OPs on the survival of UV-irradiated HaCaT cells. OPs might thus exert an anti-photoaging role by inhibiting-galactosidase activity. One manifestation of photoaging in HaCaT cells is a marked increase in the percentage of senescent cells. OPs show general anti-aging effects and, consistently, anti-photoaging effects. In addition, the toxic effect of UV-irradiation on cells does not cause immediate cell death, but forces cells into senescence, thereby inhibiting cell proliferation. Such a mechanism could explain delayed UV-induced toxicity.

The biggest difference in appearance between photoaging skin and normal skin is the wrinkling of photoaging skin due to the degradation and loss of collagen fibers and elastin [35]. MMP-1 plays a key role in degrading collagen [36]. Expression of PINP, a precursor of collagen, in OP-treated cells was increased compared with UV-irradiated cells and became more similar to controls. Thus, OP5-3 components largely reversed the increase in MMP-1 and the decrease in PINP expression caused by UV-irradiation. Such effects reflect the potential of OPs to protect against skin photoaging.

Synthetic peptides are important for the study of peptide action. These peptides address problems of insufficient sample size and sample homogeneity. The six peptides selected based on mass spectrometry also inhibited MMP-1 expression, and AIVAEVNEAAK showed the greatest effect. The interaction between AIVAEVNEAAK and MMP-1 was simulated by molecular docking. We found significant hydrogen bonding of AIVAEVNEAAK to amino acid residues at the MMP-1 active site. These bonds show small binding distances and strong binding forces. Moreover, AIVAEVNEAAK is matched well with the protein pocket, showing a good fit for each part of the peptide in the pocket groove. AIVAEVNEAAK might inhibit increased expression of MMP-1 and competitively inhibit MMP-1 binding to its ligand, thus inhibiting the ability of MMP-1 to degrade collagen. These actions could underlie the anti-photoaging effect. AIVAEVNEAAK alleviates the toxic effects of UV-irradiation on HaCaT cells and displays potential as a skin anti-photoaging agent.

In conclusion, the novel active peptides identified in this study show a prominent activity in resisting photo-oxidation. These discoveries implicate the potential of novel active peptides in the protection of skin disease.

## 4. Materials and Methods

### 4.1. Preparation of Oyster Peptides

**Figure 15:**
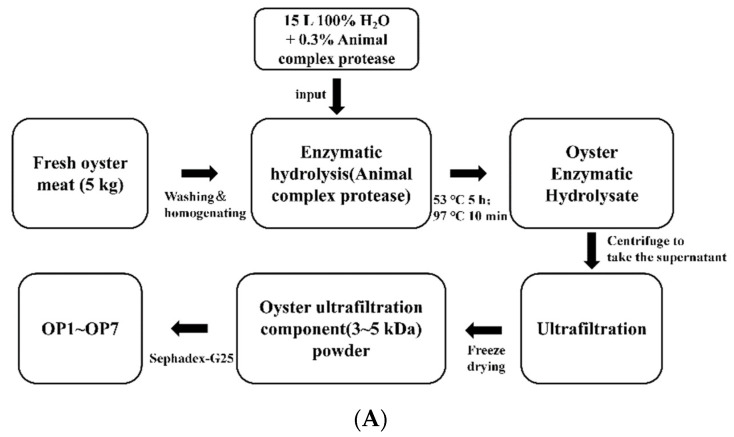

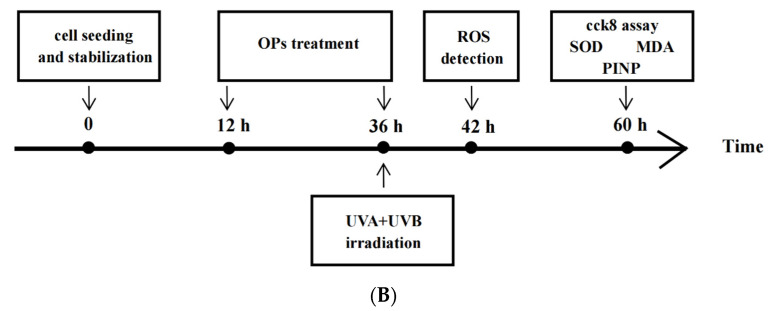
Process flow for extracting oyster peptides (OP) and schematic diagram of the experimental design for examining anti-photoaging effects in UV-irradiated HaCaT cells. (**A**) OP extraction process. Oyster enzymatic hydrolysate was successively fractionated by ultrafiltration (3~5 kDa) and Gel chromatography (**B**) Timeline of UV-irradiation and Ops (OP3, OP4, and OP5) treatment of HaCaT cells. HaCaT cells were treated with OPs starting one day before UV-irradiation. HaCaT cells were evaluated by CCK-8 assay, and measurements of ROS, SOD detection, and MDA one day after UV-irradiation.

Hong Kong oysters (C. hongkongensis) were purchased at Dongfeng Aquatic Products Market in Zhanjiang City, China. After removing all visible impurities and debris, fresh oyster meat (5 kg) was homogenized. The homogenate was diluted with three volumes of distilled water, and the homogeneous mixture was hydrolyzed for 5 h at 53 °C (pH = 7.0) by the addition of 0.3% animal complex protease (Pangbo Biotech, Nanning, China). Protease digestion was stopped and the enzymatic hydrolysate was centrifuged at 12,000 rpm for 20 min. The supernatant was sequentially fractionated by 8 kDa, 5 kDa, and 3 kDa ultrafiltration membranes (Amicon^®^ Ultra-15 centrifugal filter units, Billerica, MA, USA) to separate OUP. Finally, freeze-dried oyster peptides were further divided into seven independent components (OP1 to OP7) on a Sephadex-G25 column (Figure 15A). Protein concentration and antioxidant capacity (ABTS, OH, DPPH) were determined in vitro for each fraction with assay kits (Solarbio., Beijing, China).

### 4.2. Cell Culture

Immortalized human keratinocytes, HaCaT cells, were purchased from FuHeng Biology (Shanghai, China). Cells were cultured in Dulbecco’s modified Eagle’s medium (DMEM; Gibco, Thermo Fisher Scientific, Waltham, MA, USA) supplemented with 10% heat-inactivated fetal bovine serum (FBS; Gibco) and 1% antibiotics [100 U/mL penicillin and 100 µg/mL streptomycin (Invitrogen, Thermo Fisher Scientific, Waltham, MA, USA)] at 37 °C in a humidified 5% CO_2_ incubator (SANYO, Ōsaka, JPN). HaCaT cells were subcultured when they reached 80% to 90% confluence. Cells were cultured for 12 h in 10% heat-inactivated FBS before being used for experiments. When required, HaCaT cells were treated with OPs in a serum-free medium and incubated under the same conditions (Figure 15B).

### 4.3. UV-Irradiation of Cells

For UV-irradiation (Combined UVA and UVB irradiation), HaCaT cells were cultured in 6-well plates (3 × 10^5^ cells/well), 12-well plates (1 × 10^5^ cells/well), and 96-well plates (1 × 10^4^ cells/well) for one day. When cells sufficiently adhered, the medium was removed and cells were washed twice with PBS. Each well contained a thin layer of PBS to keep cell surfaces from drying. The lids of culture plates were opened, and the plates were placed under a UV lamp emitting both UVA (365 nm) and UVB (310 nm) light for 8 min. Irradiation dose was calculated as: Dose (mJ/cm^2^) = Exposure time (s) × Intensity (mW/cm^2^). After irradiation, cells were washed with warm PBS and incubated with serum-free DMEM for one day or longer. When appropriate, HaCaT cells were treated with OPs in a serum-free medium and were incubated under the same conditions (Figure 15B).

### 4.4. Cell Viability Assay

Cell viability was evaluated by measuring the dehydrogenase-catalyzed formation of a yellow dye in living cells. In the presence of electronic coupling reagents, WST-8 (tetrazolium salt 2-(2-methoxy-4-nitrophenyl)-3-(4-nitrophenyl)-5-(2,4-disulfophenyl) -2H-tetrazolium, monosodium salt (CCK-8)) is reduced to formazan, a yellow dye, by dehydrogenase. The amount of formazan produced catalyzed by dehydrogenase shows a direct linear relationship with the number of living cells, and can be detected by the colorimetric method.

Cells were seeded on 96-well plates at a density of 1 × 10^4^ cells/well; viability was measured 24 h after UV-irradiation. At this time, a CCK-8 stock solution (10 µL/well in PBS) was added to each well and left to incubate at 37 °C for 1 h. Absorbance was measured using a microplate reader (Varioskan Flash; Thermo, Waltham, MA, USA) at λ = 450 nm. For each sample, two experiments were performed in quadruplicate; data were averaged and presented as the percentage of viable cells with respect to untreated controls (100%). Cytotoxicity and optimal concentrations of OPs were determined using similar steps.

### 4.5. Determination of SOD, MDA, PINP, and MMP-1 Intracellular or Extracellular

Intracellular SOD activity and MDA concentration extracellular PINP and MMP-1 were measured to assess the ability to resist photo-oxidation. SOD and MDA were estimated with assay kits (Solarbio., Beijing, China). HaCaT cells in the logarithmic growth phase were seeded into a 6-well plate at 3 × 10^5^ cells/well, following operations are shown in Figure 15B. The cells were digested with trypsin and centrifuged (1000 rpm, 5 min). Then, 0.5 mL of PBS was added into the precipitated cells and the cells were cracked by an ultrasonic method in the ice water bath (300 W power, 3–5 s each time, 30 intervals, 10 s each interval), the supernatant was obtained by centrifugation (10,000 rpm, 20 min) for detecting SOD and MDA.

The ability of SOD was determined by the WST-8 method with the following principle: WST-8 can react with the superoxide anion (O_2_^−^) catalyzed by Xanthine Oxidase (XO) to produce a water-soluble formazan dye. Since SOD can inhibit the above reaction by dismutation of the catalytic O_2_^−^, the activity of SOD is negatively correlated with the amount of formazan dye production so that the enzyme viability of SOD can be calculated by colorimetric analysis of the WST-8 production.

Briefly, 1.5 mL SOD detection buffer was mixed with 800 μL WST-8 and 100 μL enzyme solution to prepare the working solution. Next, 20 μL supernatant was collected from each sample. The supernatant was then mixed with 160 μL working solution and 20 μL reaction buffer. The blank1 solution was prepared by mixing 20 μL SOD detection buffer, 160 μL working solution, and 20 μL reaction buffer. The blank2 solution consisted of 40 μL SOD detection buffer. Each mixture was incubated at room temperature for 30 min, and then the OD450 value was detected using a microplate reader. The suppression ratio was calculated using the equation:

suppression ratio = (A _blank1_—A _samples_) ÷ (A _blank1_—A _blank2_) × 100%

The SOD level was calculated using the equation:

y = 0.0106x + 0.01

where x is 1/SOD level and y is 1/suppression ratio.

Lipid peroxidation levels in HaCaT cells were observed by measuring MDA levels. MDA in acid and high temperature can condense with thiobarbituric acid (TBA) to produce brown-red 3,5,5-Trimethyloxazolidine-2,4-dione, with a maximum absorption wavelength of 532 nm. The content of peroxidation lipid in the sample can be estimated after the colorimetric analysis.

First, the detection reagent for the MDA test was prepared following the manufacturer’s instructions. Then, the supernatants from each group obtained above and the detection reagent were added according to the manufacturer’s instructions. Next, the mixtures were heated to 100 °C for 60 min and then cooled to room temperature. The mixtures were then centrifuged at 8000 *g* for 10 min. 200 μL of supernatant was added to the 96-well plate, and the absorbance of each well was measured at 450 nm, 532 nm, and 600 nm with a microplate reader. Each sample was run in duplicate, and each experiment was repeated at least three times. The content was calculated using the equation:

MDA content (nmol/mg prot) = (12.9 × (A_1_ − A_2_)—2.58 × A_3_) ×V_total_ ÷ (Cpr × V_sample_)

A_1:_ A_532_ ^sample^–A_532_ ^blank^

A_2:_ A_600_ ^sample^–A_600_ ^blank^

A_3:_ A_450_ ^sample^–A_450_ ^blank^

PINP and MMP-1 levels were measured in cell culture media using enzyme-linked immunoassay (ELISA) kits (Yutong Biological Technology Co., Changzhou, China). All experiments were conducted according to the manufacturer’s instructions. Purified Human MMP-1 antibody is coated in microtiter plate wells, making solid-phase antibodies. Then add the sample to the wells, a Combined antibody which With HRP labeled, become antibody-antigen-enzyme-antibody complex, after washing Completely, Add TMB substrate solution, TMB substrate becomes blue color At HRP enzyme-catalyzed, the reaction is terminated by the addition of a sulphuric acid solution and the color change is measured spectrophotometrically at a wavelength of 450 nm. The concentration of MMP-1 in the samples is then determined by comparing the O.D. of the samples to the standard curve. The content of PINP was determined on the same principles.

### 4.6. Evaluation of Intracellular ROS Levels

Levels of intracellular ROS were assessed using the oxidation of DCF (Sigma-Aldrich, St. Louis, MO, USA) with Leica DMIRB. DCFH-DA diffuses through the cell membrane and is enzymatically hydrolyzed by intracellular esterase to nonfluorescent DCFH which cannot diffuse through the cell membrane. Intracellular ROS subsequently oxidizes DCFH to fluorescent 2′-7′-dichlorofluorescein (DCF). The fluorescence intensity of DCF is directly proportional to the levels of intracellular ROS. HaCaT cells were seeded in 24-well plates and incubated for 12 h at 37 °C with DMEM and 5% CO_2_ in a humidified incubator, after incubation, OPs were added to the cells at 25 and 100 μg/mL, followed by further incubation for 24 h. The cells received optical treatment at a dose of 2.4 mJ/cm^2^ UVB and 28.8 J/cm^2^ UVA for 8 min. The cells were cultured in a serum-free medium for 6 h, after removal of the supernatant, five nM/well of DCFH-DA was added and cells were incubated in a humidified atmosphere (5% CO_2_, 37 °C) for 40 min then washed three times in PBS. Finally, the fluorescence intensity of DCF was detected under an inverted fluorescence microscope in the dark.

### 4.7. SA-β-Gal Staining

SA-*β*-Gal Staining using a kit, following the manufacturer’s instructions (Beyotime Biotechnology; Shanghai, China). Senescent cells usually become larger and express *β*-Gal with high enzymatic activity at pH 6.0. X-Gal, as a substrate, is catalyzed by *β*-Gal to generate dark blue products, which can be viewed directly visualized by microscopy. The operation is consistent with previous ROS assay, after removal of the supernatant, cells were washed in phosphate-buffered saline, fixed for 15 min (room temperature) in 2% formaldehyde/0.2% glutaraldehyde. Cells were then washed and incubated at 37 °C with SA-*β*-gal staining solution overnight. Staining could be directly observed with an ordinary optical microscope and was evident within 2–4 h.

### 4.8. Reversed-Phase High-Performance Liquid Chromatography (RP-HPLC) Analysis and Mass Spectroscopy

OP5 was further analyzed by RT-HPLC. The liquid chromatographic conditions were taken from Peng et al. with appropriate modifications using an Agilent 1260 chromatography system, Palo Alto, CA, USA—column: C18 (Waters, 300 Å, 3.5 µm, 4.6 mm × 150 mm, 1/pk); flow phase A—0.1% trifluoroacetic acid (TFA); flow phase B—99.9% acetonitrile and 0.1% TFA; gradient—0 to 12 min (99.9%A, 0.1%B); 12 to 15 min (99.8%A, 0.2%B), 15 to 18 min (90%A, 10%B), 18 to 23 min (85%A, 15%B); 23 to 26 min (80%A, 20%B), 26 to 30 min (100%A, 0%B); sample delivery—100 µL; flow speed 0.5 mL/min; column temperature –30 °C. Each fraction was collected separately, freeze-dried, and used in cell culture experiments to measure MMP-1 and PINP expression.

The fraction with the highest activity was sent for sequence analysis using Q Exactive (Thermo Fisher Scientific, Waltham, MA, USA) paired with Thermo U3000 HPLC (Thermo Fisher Scientific, Waltham, MA, USA). Samples were eluted using 98% H_2_O, 2% CAN, 0.1% FA (solvent A) and 98% CAN, 2% H_2_O, 0.1% FA (solvent B) at a constant flow rate of 400 nL/min. Solvent gradients are provided in Table 4. Peptide sequences were identified by electrospray ionization mass spectrometry and tandem mass spectrometry (ESI-MS/MS) (Thermo Fisher Scientific, Waltham, MA, USA) in positive ion mode. After chromatography, ESI-MS/MS was carried out using a Q Exactive™ triple quadrupole instrument (Thermo Fisher Scientific, Waltham, MA, USA) equipped with an ESI source. Sequences of characteristic peptides were determined by analysis and comparison with secondary fragments of peptides from the collision-induced dissociation spectrum of the protonated molecule [M + H]^+^ in the Uniprot database. Conditions for mass spectroscopy were: spray voltage: 2.0 kV, scanning time: 0–65 min, capillary temperature: 320 °C, resolution: 70,000, AGC target: 1 × 10 ^5^, maximum IT: 100 ms. Sequences were searched in the NCBI protein database to match parent proteins. Peptide sequences were selected, based on score and hydrophobicity, for solid-phase synthesis for verification of bioactivity.

### 4.9. Solid-Phase Synthesis of Peptides

Solid-phase synthesis of six peptides was accomplished by Qiangyao Biotechnology Co., Ltd. (Shanghai, China). The resultant peptides were assessed for purity using RP-HPLC in combination with ESI/MS (Thermo Fisher Scientific, Waltham, MA, USA).

### 4.10. Molecular Docking

Molecular docking is a computational method for the prediction of the binding of ligands to receptor binding sites. This method assesses varying positions and conformations of the ligand keeping the receptor site rigid. Molecular docking investigation was used to evaluate anti-photoaging mechanisms of action of one synthetic peptide. Peptide was the most potent of the six peptides and was selected as a ligand. MMP-1 and PINP were chosen as receptors. The stereo structure of peptide was obtained using PEP-FOLD3 software (https://bioserv.rpbs.univ-paris-diderot.fr/services/PEP-FOLD3/, accessed on 11 December 2021), and the stereo structure of MMP-1 and PINP were obtained using RCSB. Molecular docking used the Glide module in Schrodinger Maestro software (Maestro 11.9, Schrodinger, NYC, NY, USA).

### 4.11. Statistical Analysis

All quantitative data are presented as mean ± standard (mean ± S. D) deviation. Data were analyzed by one-way ANOVA. A value of *p* ≤ 0.05 was considered to be statistically significant. All analyses were performed using Statistical Analysis Software (SPSS 26.0, IBM SPSS, Armonk, NY, USA).

## 5. Conclusions

Our findings confirm that OPs pretreatment protects HaCaT cells exposed to UV-irradiation against photoaging. This activity is associated with anti-oxidant defenses, promotion of cell proliferation, inhibition of β-galactosidase, mitigation of cellular senescence of cell, and inhibition of MMP-1 expression. We report for the first time several novel peptide sequences with anti-photoaging properties. These peptides were isolated and identified from an oyster enzymatic hydrolysate.

## Figures and Tables

**Table 1:** DPPH radical, hydroxyl radical, and ABTS radical scavenging activities of OP fractions.

Group	Protein Content (%)	ABTS Free Radical Scavenging Rate (%)	DPPH Free Radical Scavenging Rate (%)	Hydroxyl Free Radicals Scavenging Rate (%)
OP1	20.20	3.00 ^d^	37.64 ^b^	59.89 ^d^
OP2	15.07	1.97 ^d^	89.49 ^a^	74.31 ^a^
OP3	11.86	11.89 ^c^	87.99 ^a^	68.14 ^b^
OP4	19.98	26.53 ^b^	12.40 ^c^	-
OP5	96.71	11.81 ^c^	7.39 ^c^	39.03 ^e^
OP6	34.12	26.10 ^b^	-	62.96 ^c^
OP7	16.37	36.61 ^a^	5.81 ^c^	60.07 ^d^

Different letters indicate significant differences between the two in the same column, *p* < 0.05; The “-” representative was not detected.

**Table 2:** Main peptide sequences analysis from OP5-3.

Sequence	Amino Sequence of OPs	Molecular Mass/Da	Number of Amino Acids
**1**	AIVAEVNEAAK	1113.6030	11
**2**	IGGIGTVPVGR	1024.6030	11
**3**	TALAIDAIINQK	1269.7292	12
**4**	AGIDQAIAR	913.4981	9
**5**	VLVPTQEAVQK	1210.6921	11
**6**	NARNAHEIEIK	1293.6789	11
**7**	TITLEVEPSDTIENVK	1786.9200	16
**8**	GVAMNPVDHPHGGGEGR	1685.7693	17
**9**	YEDQIGIR	992.4927	8
**10**	LICIVPK	841.5095	7
**11**	VTDVEIAEVLSK	1301.7078	12
**12**	VLSLDLGALVAGAK	1325.7918	14
**13**	AAVEEGVVPGGGVALVR	1578.8730	17
**14**	MYLL->M<-EKQHNR	1477.7169	11
**15**	->M<-YLL->M<-EKQHNR	1493.7119	11
**16**	LERGKLDPK	1054.6135	9
**17**	DDLVIGSPFASVK	1346.7082	13

**Table 3:** Main peptide sequence analysis from OP5-3.

Number	Amino Sequence	Hydropathicity/Hydrophobicity	Molecular Mass/Da	Number of Amino Acids
Peptide I	AIVAEVNEAAK	----+-+--+	1113.6062	11
Peptide II	IGGIGTVPVGR	-++-++-+-+	1024.6058	11
Peptide III	TALAIDAIINQK	+----+---+++	1269.7335	12
Peptide IV	VLVPTQEAVQK	---++++--++	1210.6921	11
Peptide V	GVAMNPVDHPHGGGEGR	+---++-++++++++++	1685.7718	17
Peptide VI	LICIVPK	--+--++	841.5039	7

**Table 4:** HPLC parameter setting.

Column	Trap Column: Acclaim PePmap 100, 75 μm × 2 cm, nanoviper, C18, 3 μm, 100 Å Analytical Column: C18 (L), 5 μm, 150 Å
**Chromatographic Gradient**	
Time	Phase B concentration (%)
0	5
6	8
6.5	10
45	24
51	40
54	80
59	80
59.9	5
65	5

## Data Availability

Data are available upon request.

## References

[1] Yaar M., Gilchrest B.A. (2007). Photoageing: Mechanism, prevention and therapy. Br. J. Dermatol..

[2] Sorrentino J.A., Krishnamurthy J., Tilley S., Alb J.G., Burd C.E., Sharpless N.E. (2014). p16INK4a reporter mice reveal age-promoting effects of environmental toxicants. J. Clin. Investig..

[3] Schuch A.P., Moreno N.C., Schuch N.J., Menck C.F.M., Garcia C.C.M. (2017). Sunlight damage to cellular DNA: Focus on oxidatively generated lesions. Free Radic. Biol. Med..

[4] Liu N., Matsumura H., Kato T., Ichinose S., Takada A., Namiki T., Asakawa K., Morinaga H., Mohri Y., De Arcangelis A. (2019). Stem cell competition orchestrates skin homeostasis and ageing. Nature.

[5] De Laat A.T.J., van der A.R.J., Allaart M.A.F., van Weele M., Benitez G.C., Casiccia C., Paes Leme N.M., Quel E., Salvador J., Wolfram E. (2010). Extreme sunbathing: Three weeks of small total O3columns and high UV radiation over the southern tip of South America during the 2009 Antarctic O3hole season. Geophys. Res. Lett..

[6] Kostyuk V., Potapovich A., Albuhaydar A.R., Mayer W., De Luca C., Korkina L. (2018). Natural Substances for Prevention of Skin Photoaging: Screening Systems in the Development of Sunscreen and Rejuvenation Cosmetics. Rejuvenation Res..

[7] Vayalil P.K., Mittal A., Hara Y., Elmets C.A., Katiyar S.K. (2004). Green tea polyphenols prevent ultraviolet light-induced oxidative damage and matrix metalloproteinases expression in mouse skin. J. Investig. Dermatol..

[8] Zhang H., He M. (2020). The role of a new insulin-like peptide in the pearl oyster *Pinctada fucata martensii*. Sci. Rep..

[9] Siregar A.S., Nyiramana M.M., Kim E.J., Shin E.J., Woo M.S., Kim J.M., Kim J.H., Lee D.K., Hahm J.R., Kim H.J. (2020). Dipeptide YA is Responsible for the Positive Effect of Oyster Hydrolysates on Alcohol Metabolism in Single Ethanol Binge Rodent Models. Mar. Drugs.

[10] Li Y., Qiu W., Zhang Z., Han X., Bu G., Meng F., Kong F., Cao X., Huang A., Feng Z. (2020). Oral oyster polypeptides protect ovary against d-galactose-induced premature ovarian failure in C57BL/6 mice. J. Sci. Food Agric..

[11] Zhang X., Peng Z., Zheng H., Zhang C., Lin H., Qin X. (2021). The Potential Protective Effect and Possible Mechanism of Peptides from Oyster (*Crassostrea hongkongensis*) Hydrolysate on Triptolide-Induced Testis Injury in Male Mice. Mar. Drugs.

[12] Siregar A.S., Nyiramana M.M., Kim E.J., Cho S.B., Woo M.S., Lee D.K., Hong S.G., Han J., Kang S.S., Kim D.R. (2021). Oyster-Derived Tyr-Ala (YA) Peptide Prevents Lipopolysaccharide/D-Galactosamine-Induced Acute Liver Failure by Suppressing Inflammatory, Apoptotic, Ferroptotic, and Pyroptotic Signals. Mar. Drugs.

[13] Fuda H., Watanabe M., Hui S.P., Joko S., Okabe H., Jin S., Takeda S., Miki E., Watanabe T., Chiba H. (2015). Anti-apoptotic effects of novel phenolic antioxidant isolated from the Pacific oyster (*Crassostrea gigas*) on cultured human hepatocytes under oxidative stress. Food Chem..

[14] Wang Q., Li W., He Y., Ren D., Kow F., Song L., Yu X. (2014). Novel antioxidative peptides from the protein hydrolysate of oysters (*Crassostrea talienwhanensis*). Food Chem..

[15] Wu S., Huang X. (2017). Preparation and antioxidant activities of oligosaccharides from *Crassostrea gigas*. Food Chem..

[16] Xiang N., Zhao C., Diao X., Han Q., Zhou H. (2017). Dynamic responses of antioxidant enzymes in pearl oyster Pinctada martensii exposed to di(2-ethylhexyl) phthalate (DEHP). Environ. Toxicol. Pharmacol..

[17] Qian B., Zhao X., Yang Y., Tian C. (2020). Antioxidant and anti-inflammatory peptide fraction from oyster soft tissue by enzymatic hydrolysis. Food Sci. Nutr..

[18] Xiang X.W., Zheng H.Z., Wang R., Chen H., Xiao J.X., Zheng B., Liu S.L., Ding Y.T. (2021). Ameliorative Effects of Peptides Derived from Oyster (Crassostrea gigas) on Immunomodulatory Function and Gut Microbiota Structure in Cyclophosphamide-Treated Mice. Mar. Drugs.

[19] Hwang D., Kang M.J., Jo M.J., Seo Y.B., Park N.G., Kim C.D. (2019). Anti-Inflammatory Activity of beta-thymosin Peptide Derived from Pacific Oyster (Crassostrea gigas) on NO and PGE(2) Production by Down-Regulating NF-kappaB in LPS-Induced RAW264.7 Macrophage Cells. Mar. Drugs.

[20] Sotiropoulou G., Zingkou E., Pampalakis G. (2021). Redirecting drug repositioning to discover innovative cosmeceuticals. Exp. Dermatol..

[21] Han J.H., Bang J.S., Choi Y.J., Choung S.Y. (2019). Anti-melanogenic effects of oyster hydrolysate in UVB-irradiated C57BL/6J mice and B16F10 melanoma cells via downregulation of cAMP signaling pathway. J. Ethnopharmacol..

[22] Peng Z., Chen B., Zheng Q., Zhu G., Cao W., Qin X., Zhang C. (2020). Ameliorative Effects of Peptides from the Oyster (Crassostrea hongkongensis) Protein Hydrolysates against UVB-Induced Skin Photodamage in Mice. Mar. Drugs.

[23] Buonocore G., Perrone S., Tataranno M.L. (2010). Oxygen toxicity: Chemistry and biology of reactive oxygen species. Semin. Fetal Neonatal Med..

[24] Kammeyer A., Luiten R.M. (2015). Oxidation events and skin aging. Ageing Res. Rev..

[25] Williams D.E., Andersen R.J. (2020). Biologically active marine natural products and their molecular targets discovered using a chemical genetics approach. Nat. Prod. Rep..

[26] Zhang G., Fang X., Guo X., Li L., Luo R., Xu F., Yang P., Zhang L., Wang X., Qi H. (2012). The oyster genome reveals stress adaptation and complexity of shell formation. Nature.

[27] Zarkovic N. (2020). Roles and Functions of ROS and RNS in Cellular Physiology and Pathology. Cells.

[28] Munoz-Espin D., Serrano M. (2014). Cellular senescence: From physiology to pathology. Nat. Rev. Mol. Cell Biol..

[29] McHugh D., Gil J. (2018). Senescence and aging: Causes, consequences, and therapeutic avenues. J. Cell Biol..

[30] Tchkonia T., Kirkland J.L. (2018). Aging, Cell Senescence, and Chronic Disease: Emerging Therapeutic Strategies. JAMA.

[31] Calcinotto A., Kohli J., Zagato E., Pellegrini L., Demaria M., Alimonti A. (2019). Cellular Senescence: Aging, Cancer, and Injury. Physiol. Rev..

[32] Saleh T., Tyutyunyk-Massey L., Murray G.F., Alotaibi M.R., Kawale A.S., Elsayed Z., Henderson S.C., Yakovlev V., Elmore L.W., Toor A. (2019). Tumor cell escape from therapy-induced senescence. Biochem. Pharmacol..

[33] Shmulevich R., Krizhanovsky V. (2021). Cell Senescence, DNA Damage, and Metabolism. Antioxid. Redox Signal..

[34] Hernandez-Segura A., Nehme J., Demaria M. (2018). Hallmarks of Cellular Senescence. Trends Cell Biol..

[35] Shin J.W., Kwon S.H., Choi J.Y., Na J.I., Huh C.H., Choi H.R., Park K.C. (2019). Molecular Mechanisms of Dermal Aging and Antiaging Approaches. Int. J. Mol. Sci..

[36] Van Doren S.R. (2015). Matrix metalloproteinase interactions with collagen and elastin. Matrix Biol..

